# Impact of a Mobile Nutrition App on Dietary Outcomes in Cancer Survivors: Pilot Feasibility Study

**DOI:** 10.2196/79215

**Published:** 2026-03-31

**Authors:** Sang Won Park, Woo Jin Kim, Oh Beom Kwon, Inhyeok Yim

**Affiliations:** 1 Institute of Clinical Research, Biolink Inc, Daegu, Republic of Korea; 2 Department of Pulmonary Medicine, Kangwon National University, Chuncheon, Gangwon-do, Republic of Korea; 3 Department of Family Medicine, Kangwon National University Hospital, 156, Baengnyeong-ro, Chuncheon, Gangwon-do, 24289, Republic of Korea, 82 1092395470

**Keywords:** mobile health, cancer, nutrition, digital health, quality of life, mobile phone

## Abstract

**Background:**

Cancer survivors frequently face persistent nutrition-related challenges after treatment. Mobile health tools may extend access to dietary self-management support beyond clinic settings, but feasibility and preliminary effects remain insufficiently characterized in this population.

**Objective:**

This study aims to evaluate the feasibility, user engagement, and preliminary effects of a 4-week mobile nutrition app on dietary behavior and quality of life (QoL) among cancer survivors, and to explore whether higher engagement is associated with greater improvements.

**Methods:**

A single-arm, prospective pilot feasibility study was conducted at a tertiary cancer center in Korea. Participants used a mobile nutrition app that provided dietary feedback and self-monitoring features. In-app log data were analyzed to determine engagement metrics (session frequency, duration, and gap regularity) using an elbow-based 10-minute session threshold. Primary outcomes included the Nutrition Quotient for Adults (NQ-2021) and European Organization for Research and Treatment of Cancer Quality of Life Questionnaire Core 30 (EORTC QLQ-C30) scores, measured before and after 4 weeks. Nonparametric paired analyses assessed changes, and exploratory correlations examined relationships between engagement and outcomes.

**Results:**

Among 27 enrolled participants, 24 cancer survivors (88.9%) completed the intervention and postassessment; the majority were female (17/24, 70.8%), with a mean age of 58.5 (SD 8.7) years. Breast cancer was the most common diagnosis (11/24, 45.8%), and most participants reported no diet-related adverse effects (20/24, 83.3%) and stable body weight during the study period. Participants averaged 2.3 app sessions per day and a median cumulative use of 177.5 minutes. Retention was 88.9%, and median adherence to daily self-monitoring exceeded 85%. The NQ Moderation domain improved significantly (mean 76.6, SD 17.5 → mean 81.0, SD 13.7; *P*=.02), while Balance and Dietary Behavior showed positive trends (mean 63.7, SD 16.1 → mean 65.5, SD 13.3, *P*=.14; mean 64.9, SD 17.3 → mean 67.1, SD 15.9, *P*=.10). In QoL outcomes, appetite loss decreased (mean 17.9, SD 22.0 → mean 7.7, SD 14.6; *P*=.03) and global health status increased modestly (mean 68.5, SD 21.4 → mean 72.9, SD 20.1; *P*=.08). Higher engagement correlated with improved moderation (*r*=0.46; *P*=.02) and reduced appetite loss (*r*=–0.42; *P*=.04). Exploratory subgroup analyses suggested stronger effects among participants aged 60 years and older (ΔNQ Moderation +7.9; *P=*.04) and those with longer cancer survivorship (>3 years; *P=*.047). No adverse events were reported.

**Conclusions:**

This pilot feasibility study demonstrates high user engagement, satisfactory retention, and preliminary improvements in nutritional behavior and QoL among cancer survivors using a mobile nutrition app. These findings indicate the feasibility of a larger controlled trial to confirm the app’s effectiveness and explore long-term adherence strategies.

## Introduction

Poor nutritional status is a highly prevalent and serious problem in cancer care, affecting 40%‐80% of patients depending on cancer type and treatment phase [1,2]. This high prevalence reflects both tumor-related metabolic alterations and treatment-induced side effects, such as appetite loss or gastrointestinal dysfunction, which compromise dietary intake. Malnourished patients experience poorer treatment tolerance, higher complication rates, delayed recovery, and diminished quality of life (QoL). Poor nutritional status has also been associated with reduced survival [3,4]. Nutritional support interventions—including early dietetic counseling and individualized nutrition plans—can mitigate these effects and improve both clinical and patient-reported outcomes [1,5]. Accordingly, current oncology guidelines recommend routine nutrition screening and intervention across all phases of cancer care [2,6,7].

Despite these recommendations, access to professional dietary support remains limited. Many cancer survivors report unmet nutritional needs due to lack of referrals, time constraints, and system-level barriers. In 1 study, only 23% of survivors had consulted a dietitian after treatment, most citing difficulty accessing expert guidance [8]. Consequently, many survivors turn to unverified online information, underscoring the urgent need for accessible, evidence-based digital tools to support nutritional self-management [9]. Importantly, nutritional challenges in cancer survivorship are heterogeneous and extend beyond inadequate intake alone. While many survivors experience appetite loss and reduced dietary intake during or after treatment, others—particularly survivors of breast, colorectal, and endometrial cancers—are prone to excess weight gain or unhealthy dietary patterns that are associated with increased risks of recurrence and metabolic comorbidities. Accordingly, contemporary survivorship guidelines, including those of the American Cancer Society (ACS), emphasize not only sufficient energy and protein intake but also the adoption of balanced, high-quality dietary patterns and moderation of excessive or nutrient-poor consumption. Nutritional support in survivorship, therefore, requires an approach that addresses both insufficient intake and maladaptive overconsumption, rather than focusing solely on weight loss or caloric increase.

Mobile health (mHealth) apps represent a promising and scalable approach to extend nutrition support beyond the clinic. These apps can provide personalized dietary education, facilitate self-monitoring of food intake, and enable real-time symptom tracking beyond traditional clinical settings [9,10]. Digital symptom monitoring during active cancer treatment has been associated with improved patient outcomes—including better QoL, fewer emergency visits, and even survival benefits—by enabling earlier supportive interventions [3,11,12]. Building on these advances, early studies of mHealth-based nutrition interventions in cancer care have reported encouraging outcomes. App-based programs targeting diet or weight management have demonstrated improvements in dietary quality, weight maintenance, and patient satisfaction in small pilot trials [12-14]. For instance, a mobile app for head and neck cancer survivors showed high engagement and acceptability for diet and symptom tracking. However, despite these encouraging findings, the overall evidence base remains limited. Most prior studies have been small or short-term, with few using rigorous designs or enrolling diverse cancer populations. Moreover, the relationship between app engagement and nutritional or QoL outcomes remains largely unexplored. These gaps underscore the need for a rigorously designed pilot study to establish feasibility parameters and explore engagement-outcome relationships before a future definitive trial.

Given these knowledge gaps and the widespread nutrition challenges among cancer survivors, we developed a new mHealth-based dietary intervention. This intervention was designed to support healthy dietary pattern formation across a broad spectrum of survivorship needs, including individuals experiencing appetite loss and insufficient intake as well as those requiring dietary balance and moderation to prevent excess weight gain. Before long-term efficacy can be meaningfully evaluated, it is therefore necessary to establish whether a candidate intervention is feasible in real-world survivorship settings, whether users engage with it consistently, and whether early behavioral and symptom changes can be detected. Accordingly, we conducted a structured pilot feasibility study to evaluate this intervention with a specific focus on recruitment, retention, engagement patterns, and short-term behavioral responsiveness. The objectives were not to establish long-term efficacy but to assess feasibility and preliminary effects and to generate critical parameters—such as adherence rates, engagement intensity, and engagement-outcome associations—required to inform the design of a future randomized, long-term trial. By integrating multidimensional dietary guidance with detailed, log-based engagement analytics and validated patient-reported outcomes, this study aims to address a key methodological gap in prior mHealth nutrition research and to provide foundational evidence for the development of rigorously designed, longer-term intervention studies in cancer survivorship.

## Methods

### Study Design and Sample

This pilot study was conducted as a single-arm, prospective feasibility trial in Korea. The study followed the CONSORT (Consolidated Standards of Reporting Trials) 2010 extension for pilot and feasibility trials, with no protocol modifications or interim analyses after trial initiation [15]. To enhance completeness of descriptive reporting, we also considered selected STROBE (Strengthening the Reporting of Observational Studies in Epidemiology) items relevant to participant characteristics and outcome reporting; however, CONSORT served as the principal reporting framework for this feasibility intervention [16]. Participants were recruited from Family Medicine and Hematology-Oncology outpatient clinics of a tertiary cancer center between October and December 2024. Treating physicians identified potentially eligible cancer survivors during follow-up visits and referred them to a research nurse with more than 3 years of oncology care experience. The nurse conducted eligibility screening, obtained written informed consent, provided training on app installation and use, and monitored participants throughout the study period. Recruitment was conducted consecutively via physician referrals and informational leaflets displayed in the clinics. A single research nurse provided standardized app training and follow-up support for all participants to ensure procedural consistency. Eligible participants were adult (aged 20‐69 years) cancer survivors who had completed curative-intent treatment—surgery, chemotherapy, and/or radiotherapy for any cancer type—and owned a smartphone capable of running the study app. All participants had undergone curative surgical resection as their primary treatment and were within 5 years of completing curative therapy. Except for 4 individuals with mild physical limitations, most participants were able to perform light daily activities without restriction. Information on employment status and comorbidities was not collected in this pilot study. Key exclusion criteria were ongoing active cancer treatment or hospice or palliative care, refusal to participate, significant cognitive impairment precluding informed consent, and inability to use the smartphone app, even with caregiver assistance. Participants could withdraw at any time without penalty. Based on institutional registry data (average 2153 new cancer diagnoses annually, 2019‐2023), a simple proportion estimate with a 0.10 margin of error and 90% CI suggested a required sample of approximately 66 participants for adequate precision. However, as this pilot aimed primarily to evaluate feasibility outcomes (recruitment, adherence, and usability) rather than statistical efficacy, a pragmatic target of approximately 50 participants was set, accounting for potential attrition. A total of 27 participants were enrolled, of whom 24 completed the 28-day intervention and provided both pre- and postintervention data for analysis. Recruitment and follow-up were planned over a 4-week intervention window per participant. Feasibility endpoints included recruitment rate, retention, and adherence; these parameters were predefined to assess study progression. No outcome measures were modified after trial initiation.

### Mobile App Intervention and Data Acquisition

**Figure 1.**
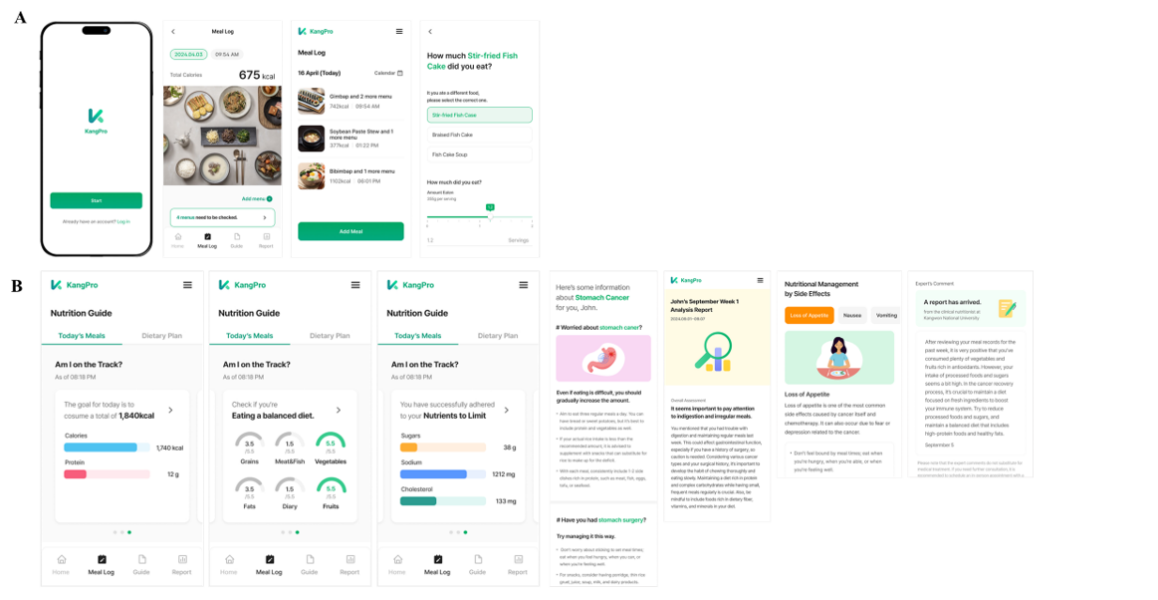
Nutrition reports by generative artificial intelligence in mobile apps for patients with cancer. (**A**) Recording meals process (**B**) Result nutrition analysis and reports by generative artificial intelligence.

Participants received access to a custom mobile app designed to support dietary management in cancer survivorship. The app was intentionally designed to support balanced, sustainable eating behaviors across heterogeneous survivorship needs—including both insufficient intake due to appetite loss and excessive or unbalanced intake associated with weight gain—rather than focusing solely on weight loss (Figure 1A). Nutrient composition and feedback were based on the Korea Food and Drug Safety Food and Nutrient Database. The app generated artificial intelligence–based nutrition reports analyzing meal composition and nutrient balance using this database (Figure 1A and B). Participants could also report symptoms, which investigators monitored in real time. The app allowed users to record their meals and dietary intake through text search or photograph uploads and to view a personalized summary of their nutritional patterns. It also automatically generated artificial intelligence–based nutrition reports that analyzed meal composition and nutrient balance using a national food composition database. The app also enabled remote symptom monitoring, as participants could report symptoms that investigators could oversee in real time.

Although the app generated quantitative dietary intake estimates for user feedback, these values were not prespecified as study end points and were therefore not analyzed for efficacy evaluation. The primary quantitative outcomes were validated questionnaire-based measures of dietary quality based on Nutrition Quotient for Adults (NQ-2021) and European Organization for Research and Treatment of Cancer Quality of Life Questionnaire Core 30 (EORTC QLQ-C30), along with app engagement metrics. Participants were instructed to use the app as desired over the 28-day study period, and weekly phone or text message check-ins by study staff were used to encourage engagement. These check-ins were standardized brief contacts focused on technical support and general encouragement, without providing individualized dietary counseling or clinical advice, and were applied uniformly to all participants. Participants who had difficulty navigating the app could be assisted by a family member or caregiver.

All user interactions with the app were automatically recorded in time-stamped log files. For data analysis, raw usage logs were filtered to include records only from valid study participants and restricted to each participant’s 28-day intervention window. An app usage session was defined as a series of consecutive interactions separated by less than 30 minutes. In other words, any app events occurring within a 30-minute interval were grouped as 1 session, and a break of 30 minutes or more initiated a new session.

### Operational Definition of App Sessions

**Figure 2.**
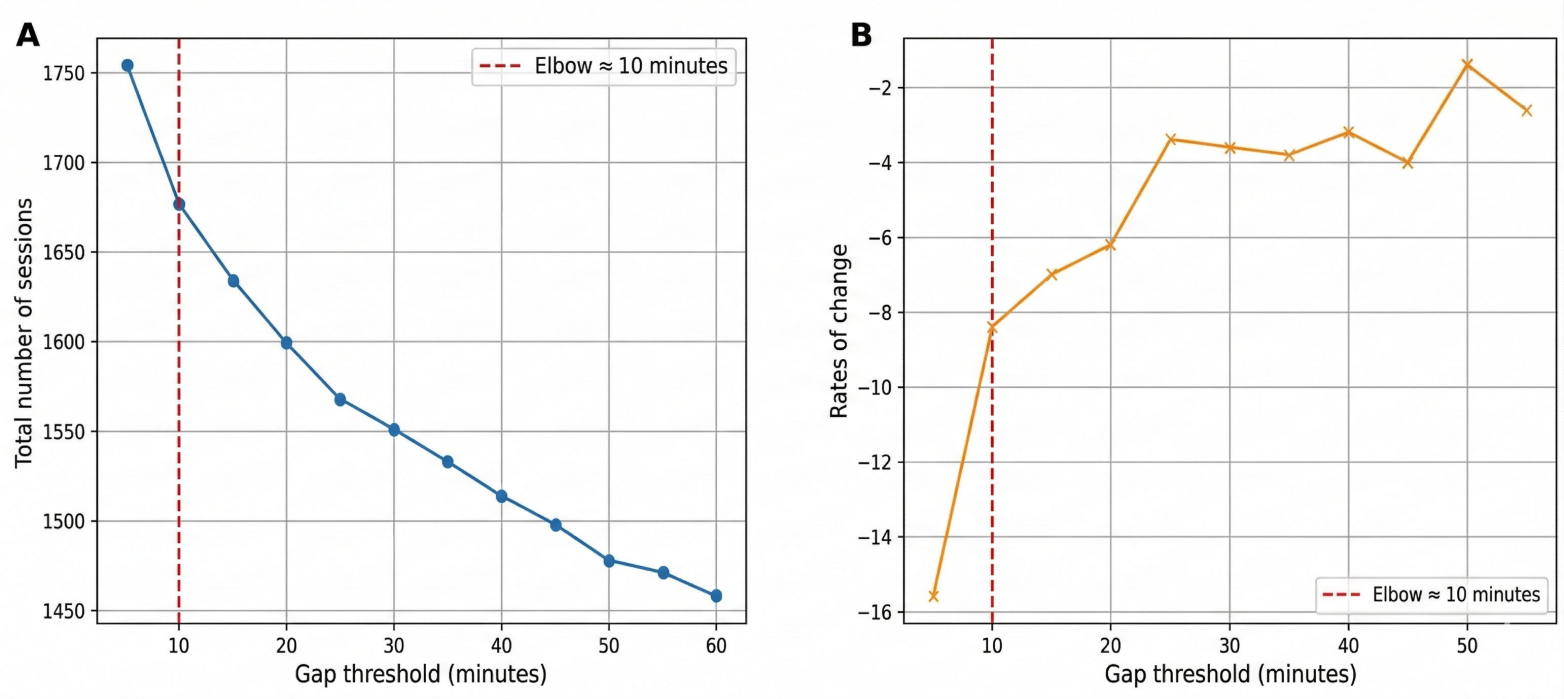
Identification of optimal session gap threshold using the elbow method. (A) Total number of app usage sessions across gap thresholds from 0 to 60 minutes. As the allowed time gap between consecutive interactions increases, separate activities are more likely to be grouped into the same session, leading to a gradual decrease in the total number of sessions. (**B**) First derivative showing rate of change in session count. The elbow point at 10 minutes (red dashed line) was selected as the optimal threshold for defining discrete app usage sessions.

To define user sessions from time-stamped login data, we applied a threshold-based approach that dynamically identifies session boundaries [17,18]. Specifically, we examined a range of time gaps (0‐60 minutes in 10-minute increments) between consecutive login events to identify an appropriate threshold for segmenting discrete usage sessions (Figure 2A and B). For each gap threshold, user activity was aggregated such that any login occurring after a period longer than the defined threshold was considered the start of a new session. This procedure was repeated for all users, and the total number of sessions was calculated for each threshold value. To identify the optimal cutoff, we analyzed the rate of change in total session counts across thresholds by calculating the first derivative. This approach quantified how quickly session counts stabilized as the threshold increased. The elbow point—where increasing the threshold produced only minimal further reduction in the total session count—was interpreted as the balance between splitting continuous usage into too many fragments and merging clearly separate sessions into one. This inflection occurred at a 10-minute gap, which we therefore adopted as the operational session boundary. In simple terms, very small gaps overdivide normal continuous use, while very large gaps merge independent activities into a single session; the 10-minute “elbow” provides the most stable midpoint between these extremes. This data-driven procedure provides an objective and reproducible threshold, analogous to the elbow method commonly used in clustering analysis [19]. Our approach further aligns with prior studies on user sessionization heuristics, which typically use gap-based thresholds (around 25‐30 minutes) to segment user activity into meaningful behavioral units [20].

### QoL Assessment

QoL was measured using the EORTC QLQ-C30 (version 3.0) [21]. The survey for EORTC QLQ-C30 was administered before and after app use following a detailed, face-to-face explanation of the survey procedures provided by a specialized research nurse (SJP), with additional consultation from clinical specialists (WJK, OBK, and IHY). This validated, cancer-specific instrument includes 30 items covering 5 functional domains, 3 multi-item symptom domains, 1 global health domain, and 6 single-item symptom scales. Scores were transformed to standardized 0‐100 scales according to the EORTC scoring manual [22]; higher functional and global health scores indicated better QoL, whereas higher symptom scores reflected greater symptom burden.

### Nutrition Assessment

Dietary intake and behavior were evaluated using the NQ-2021, a nutrition assessment tool developed and validated by the Korean Nutrition Society [23]. The survey for NQ-2021 was administered before and after app use following a detailed, face-to-face explanation of the survey procedures provided by a specialized research nurse, with additional consultation from clinical specialists (WJK, OBK, and IHY). The NQ-2021 consists of 18 items in 3 domains: Balance (consumption frequency of recommended food groups), moderation (frequency of unhealthy foods or eating behaviors), and practice (healthy eating habits). Each item score is weighted according to an established algorithm, and domain scores are summed and standardized to a 0‐100 scale. Higher NQ-2021 scores reflect better overall diet quality and healthier eating behaviors.

### Statistical Analysis

Because of the behavioral nature of the intervention, neither participants nor investigators were blinded. All statistical analyses were descriptive and exploratory. All statistical analyses were descriptive and exploratory. Within-subject pre-post comparisons were conducted using the Wilcoxon signed rank test, appropriate for small samples with nonnormal distributions. Higher postintervention functional or nutritional scores were interpreted as improvement, whereas lower symptom scores indicated reduced symptom burden. Exploratory subgroup analyses examined differences in outcomes by engagement level and participant demographics.

Engagement metrics included total usage time, maximum consecutive days of app use (≥14,≥21, and ≥28 days), average sessions per day, mean intersession gap time, and the standard deviation of intersession gaps as a measure of variability. Analyses were performed on complete cases; missing data were described but not imputed. Statistical analyses were conducted using Python (version 3.9; Python Software Foundation) and R (version 4.1; R Core Team).

### Ethical Considerations

The study was approved by the Kangwon National University Hospital Institutional Review Board (KNUH-2024-07-010) and conducted in accordance with the Declaration of Helsinki and relevant Korean privacy regulations. Written informed consent was obtained from all participants. The original consent permitted secondary analyses of deidentified data without additional approval. All data were anonymized using coded identifiers and stored on encrypted, access-restricted servers. Participants received a modest gift card upon study completion, as approved by the institutional review board. No protocol modifications or interim analyses were performed after trial initiation.

## Results

### Study Population and Baseline Characteristics

A total of 24 participants completed both pre- and postintervention assessments, including 17 (70.8%) females (Table 1). Except for 4 participants with minor physical limitations, all were able to perform light daily activities without restriction. The mean age was 61.57 (SD 8.22) years for males and 55.94 (SD 8.21) years for females. Primary tumors included breast cancer (11/24, 45.83%), colorectal cancer (3/24, 12.50%), gastric cancer (3/24, 12.50%), lung cancer (3/24, 12.50%), thyroid cancer (2/24, 8.33%), lymphoma (1/24, 4.17%), and renal cancer (1/24, 4.17%). Most participants used the app for weight maintenance (n=20, 83.3%), while 4 (16.7%) aimed for weight loss. In terms of physical activity, 12 (50.0%) reported unrestricted activity, 8 (33.3%) light-intensity activity, and 4 (16.7%) a predominantly sedentary lifestyle. Meal frequency was reported as 3 times daily by 18 (75.0%) participants, twice daily by 5 (20.8%), and 4 times by 1 (4.2%). Most participants (20/24, 83.3%) were classified as not applicable with respect to diet-related adverse effects. Body weight remained stable, with a mean of 60.8 (SD 11.5) kg before and 61.0 (SD 11.4) kg after the intervention.

**Table 1.** Patient characteristics.

Variables	Values (N=24)
Sex (female), n (%)	17 (70.8)
Age (years), mean (SD)	
Male	61.57 (8.22)
Female	55.94 (8.21)
Weight (kg), mean (SD)	
Before (mobile app usage)	60.8 (11.5)
After (mobile app usage)	61.0 (11.4)
After operation, mean (SD)	3.2 (1.9)
App usage purpose for weight, n (%)	
Maintenance	20 (83.3)
Loss	4 (16.7)
Activity, n (%)	
Light-intensity physical activity	8 (33.3)
Unrestricted physical activity	12 (50.0)
Predominantly sedentary lifestyle	4 (16.7)
Meals (in a day), n (%)	
Twice	5 (20.8)
Three times	18 (75.0)
Four times	1 (4.2)
Dietary-related adverse effects, n (%)	
Not applicable	20 (83.33)
Immunosuppression and throat pain	1 (16.7)
Constipation	1 (16.7)
Multisymptomatic (constipation, xerostomia, throat pain, etc)	1 (16.7)
Xerostomia	1 (16.7)
Primary tumor, n (%)	
Thyroid	2 (8.33)
Colorectal	3 (12.50)
Lymphoma	1 (4.17)
Renal	1 (4.17)
Gastric	3 (12.50)
Breast	11 (45.83)
Lung	3 (12.50)

### Feasibility Outcomes—Engagement and App Usage Characteristics

A total of 27 participants completed baseline assessments and initiated the intervention. Of these, 24 (88.9%) participants completed the full 4-week intervention and postintervention assessments. Three participants discontinued app use within the first 2 weeks of the study and did not complete the postintervention questionnaires. All 3 noncompleters completed baseline nutrition and QoL assessments, but postintervention data were missing due to early discontinuation of app use. No partial postintervention questionnaires were recorded. Participants who did not complete the study (n=3) did not differ markedly from completers with respect to age, sex, cancer type, or baseline nutrition and QoL scores. However, all noncompleters exhibited early disengagement from app use.

Among participants who completed the intervention, using the predefined sessionization criterion, a total of 82,385 raw app interaction log entries were consolidated into 1551 distinct usage sessions across the 24 participants during the 4-week intervention period. Overall engagement with the app was high. Participants used the app a mean of 2.3 sessions per day, with a median cumulative usage time of 177.5 minutes over the intervention period. Engagement was sustained throughout the study, with no marked decline in usage over time. Adherence to daily self-monitoring was high, with a median adherence rate exceeding 85%, and 88.9% (24/27) of enrolled participants completed the full 4-week intervention, indicating satisfactory retention. Patterns of use varied between individuals, but most participants demonstrated regular engagement characterized by repeated short sessions distributed across the day, consistent with meal logging and dietary review behaviors. Analysis of engagement intensity showed that participants differed in session frequency, cumulative usage time, and regularity of use. These engagement metrics were subsequently used to stratify participants into subgroups (eg, by consecutive days of use, total usage time, daily usage frequency, and intersession gap regularity) for exploratory analyses examining associations between engagement patterns and nutritional and QoL outcomes.

### Changes in Nutrition and QoL After App Use

After the 4-week app intervention, participants showed significant improvements in specific nutritional and symptom outcomes. In particular, the nutrition quotient moderation domain score increased from 76.61 (SD 15.36) at baseline to 81.04 (SD 12.69) postintervention, a statistically significant improvement (*P=*.02) (Table 2). This indicates a meaningful reduction in the frequency of unhealthy food choices and overeating behaviors after using the app. In contrast, other nutrition domain scores—including balance (fruits or vegetables intake) and implementation (healthy eating practices)—and the overall nutrition score did not change significantly. Among QoL measures, the most notable change was a decrease in appetite loss symptoms. The mean appetite loss score improved from 17.94 (SD 22.0) (higher scores indicate worse symptoms) to 7.69 (SD 14.62) after the intervention (*P*=.03), suggesting that participants experienced significantly less loss of appetite following app-guided dietary management. Other EORTC QLQ-C30 domains, including global health status and functional scales (physical, role, emotional, cognitive, and social), showed no statistically significant pre-post differences. Symptom scales such as fatigue, pain, nausea or vomiting, insomnia, constipation, diarrhea, and financial difficulties likewise exhibited no significant changes.

**Table 2.** Comparison of nutrition and quality of life before and after app use^a^.

Characteristics	Before (n=24), mean (SD)	After (n=24), mean (SD)	*P* value
Nutrition			
Moderation	76.61 (15.36**)**	81.04 (12.69**)**	.02^a^
Balance	59.25 (16.95)	60.29 (16.04)	.20
Implementation	78.5 (13.22)	75.99 (11.74)	.88
Nutritional score	72.16 (10.63)	72.8(10.9)	.28
Quality of life			
Global health	53.2 (12.53)	47.43 (16.1)	.84
Appetite loss	17.94 (22.0**)**	7.69 (14.62**)**	.03^a^
Cognitive	82.03 (12.65)	82.05 (17.3)	.08
Emotional	78.2 (18.17)	69.87 (23.21)	.72
Social	94.87 (10.51)	76.92 (17.4)	>.99
Physical	86.16 (12.3)	80.51 (17.52)	.80
Role	83.34 (24.52)	79.49 (15.45)	.77
Nausea and vomiting	2.57 (6.27)	3.85 (9.99)	.20
Constipation	20.51 (25.6)	25.64 (33.76)	.84
Diarrhea	17.93 (17.28)	12.82 (16.88)	.67
Pain	15.38 (17.29)	12.82 (15.45)	.20
Dyspnea	23.07 (31.58)	25.64 (27.74)	.94
Insomnia	28.18 (18.49)	30.77 (21.35)	.96
Fatigue	32.45 (23.33)	42.73 (23.06)	.95
Financial difficulties	10.25 (16.0)	20.51 (21.68)	.92

a Statistically significant results (*P*<.05).

### Changes in Nutrition and QoL Based on App Usage Intensity

Greater usage intensity was generally linked to greater improvements in nutrition and QOL measures. Consecutive days of app use (Table 3 and Table S1 in Multimedia Appendix 1) showed a clear dose-response relationship with outcomes. Participants who used the app for at least 14 consecutive days showed a significant increase in their Moderation score compared with baseline (*P*=.02). Those with ≥21 consecutive days of usage had a significant improvement in their moderation domain score (*P*=.04), reflecting better intake of recommended food groups. Furthermore, participants who engaged with the app for the full 28 days experienced significant gains in the balance domain (*P*=.02) and also in physical functioning (*P*=.047)—a QoL improvement not seen with shorter usage durations.

**Table 3.** Characteristics according to consecutive days of app use^a^.

Days of app use	Before use	After use	*P* value
14 days (nutrition)			
Moderation	75.11 (17.51)	80.58 (13.74)	.02^b^
21 days (nutrition)			
Moderation	72.71 (20.14)	77.99 (14.25)	.04^b^
28 days (nutrition)			
Balance	55.8 (15.07)	65.29 (15.75)	.02^b^
28 days (quality of life)			
Physical	84.74 (10.69)	87.62 (7.13)	.047

a Results are expressed as mean (SD).

b Statistically significant results (*P*<.05).

Total accumulated usage time was another important factor (Table 4). Participants in the high total use time group (≥177.5 minutes of cumulative app use over 4 weeks) achieved significant improvements in the moderation domain and cognitive functioning scores (both *P*=.02). In contrast, those with lower total usage time (<177.5 minutes) did not show any significant changes in these outcomes, implying that a minimum threshold of engagement time may be necessary to produce measurable benefits.

**Table 4.** Comparison based on total app usage time^a^.

Characteristics	Total app use time <177.5 minutes (n=12)			Total app use time ≥177.5 minutes (n=12)		
	Before	After	*P* value	Before	After	*P* value
Nutrition						
Moderation	76.45 (16.06)	82.33 (11.48)	.08	76.77 (15.33)	79.75 (14.18)	.02^b^
Balance	56.84 (19.08)	56.66 (16.62)	.28	61.66 (14.97)	63.92 (15.26)	.28
Implementation	73.35 (10.90)	72.51 (13.05)	.60	83.65 (13.74)	79.47 (9.55)	.92
Nutritional score	69.33 (11.97)	70.70 (11.92)	.26	74.99 (8.68)	74.89 (9.84)	.48
Quality of life						
Global health	49.30 (12.02)	47.92 (13.82)	.64	56.94 (15.43)	51.39 (16.22)	.86
Appetite loss	16.66 (22.47)	8.33 (15.07)	.11	5.55 (12.96)	0.00 (0.00)	.08
Cognitive	73.59 (22.96)	72.22 (28.72)	.39	81.93 (11.13)	86.11 (9.62)	.03^b^
Emotional	68.05 (26.79)	68.06 (31.35)	.25	81.24 (15.12)	75.00 (14.65)	.91
Social	86.12 (27.36)	79.17 (25.74)	.94	91.66 (15.08)	77.78 (19.24)	.99
Physical	85.01 (10.29)	85.56 (11.31)	.53	87.77 (12.66)	83.33 (17.17)	.83
Role	88.89 (14.78)	80.56 ( 21.12)	.93	81.95 (25.07)	86.11 (15.62)	.34
Nausea and vomiting	5.57 (8.22)	4.17 (10.36)	.25	1.39 (4.82)	1.39 (4.81)	.33
Constipation	24.99 (32.18)	27.78 (37.15)	.98	27.77 (31.25)	27.78 (27.83)	.57
Diarrhea	16.65 (17.39)	11.11 (16.41)	.46	5.55 (12.96)	5.55 (12.97)	.92
Pain	12.50 (14.42)	9.72 (11.14)	.29	22.22 (23.93)	20.83 (20.26)	.25
Dyspnea	13.88 (17.15)	16.67 (22.47)	.98	19.44 (33.21)	19.44 (26.43)	.61
Insomnia	36.09 (26.44)	38.89 (23.93)	.90	24.98 (20.72)	27.78 (19.24)	.91
Fatigue	38.87 (22.98)	43.52 (28.21)	.70	25.91 (24.31)	31.48 (18.25)	.95
Financial difficulties	22.22 (32.83)	16.67 (22.47)	.50	11.10 (16.40)	19.44 (22.28)	.99

a Values are expressed as mean (SD).

b Statistically significant results (*P*<.05).

### Changes in Nutrition and QoL Based on App Usage Frequency

Daily usage frequency showed similar trends in Table 5. High-frequency users who used the app on average at least 2.5 times per day (eg, multiple log-ins or meal recordings per day) demonstrated significant improvements in various areas: their moderation and balance scores both increased significantly (*P*=.002 and .003, respectively), and their overall nutrition score (NQ total) also rose markedly (*P*=.008). Additionally, this high-frequency group showed a significant enhancement in cognitive QoL scores (*P*=.04) and pain (*P*=.047). In contrast, participants with low daily usage frequency (<2.5 uses per day) did not exhibit any statistically significant changes in nutrition scores or QoL measures. These results indicate that more frequent interaction with the app was associated with greater dietary improvements and perceived cognitive benefits.

**Table 5.** Comparison according to app usage counts in a day^a^.

Characteristics	Low frequency (<2.5 per day, n=12)			High frequency (≥2.5 per day, n=12)		
	Before	After	*P* value	Before	After	*P* value
Nutrition						
** **Moderation	75.79 (16.13)	76.21 (14.80)	.44	77.42 (15.21**)**	85.88 (8.16**)**	.002^b^
** **Balance	56.22 (19.83)	51.01 (14.48)	.90	62.28 (13.69**)**	69.58 (11.84)	.003^b^
Implementation	77.14 (12.06)	73.58 (12.21)	.84	79.87 (14.69)	78.40 (11.24)	.75
Nutritional score	70.46 (11.61)	67.60 (11.18)	.96	73.86 (9.75**)**	77.99 (8.02**)**	.008^b^
Quality of life						
Global health	50.00 (13.76)	48.61 (15.82)	.66	56.24 (14.28)	50.69 (14.42)	.80
Appetite loss	19.43 (22.28)	8.33 (15.07)	.06	2.77 (9.61)	0.00 (0.00)	.16
Cognitive	72.21 (22.83)	72.22 (27.83)	.37	83.32 (10.04**)**	86.11 (11.96**)**	.04^b^
Emotional	68.74 (26.86)	64.58 (28.67)	.76	80.55 (15.61)	78.47 (17.21)	.57
Social	86.11 (26.42)	72.22 (23.92)	.98	91.67 (16.66)	84.72 (19.41)	.97
Physical	83.34 (11.89)	78.89 (17.49)	.95	89.43 (10.43)	90.00 (7.25)	.17
Role	81.95 (25.07)	75.00 (19.46)	.95	88.89 (14.78)	91.67 (13.29)	.23
Nausea and vomiting	2.78 (6.50)	5.56 (10.86)	.64	4.17 (7.55)	N/A^c^	.04^b^
Constipation	27.77 (31.25)	33.33 (37.61)	.93	24.99 (32.18)	22.22 (25.95)	.50
Diarrhea	13.88 (17.15)	11.11 (16.41)	.76	8.32 (15.06)	5.55 (12.97)	.50
Pain	16.66 (15.88)	16.67 (15.89)	.52	18.07 (24.06**)**	13.89 (18.58)	.047^b^
Dyspnea	24.99 (32.18)	25.00 (28.87)	.74	8.32 (15.06)	11.11 (16.41)	.97
Insomnia	33.32 (28.43)	36.11 (22.29)	.89	27.76 (19.24)	30.55 (22.29)	.91
Fatigue	40.73 (28.96)	46.30 (28.76)	.83	24.05 (14.84)	28.70 (14.57)	.91
Financial difficulties	13.88 (22.28)	22.22 (25.95)	.90	19.43 (30.01)	13.89 (17.16)	.83

a Results are presented as mean (SD).

b Statistically significant results (*P*<.05).

c N/A: not applicable.

### Changes in Nutrition and QoL Based on Regular App Usage

Participants who used the app consistently throughout the day—defined as daily use with intervals of 7 hours or less (eg, morning, afternoon, and evening)—demonstrated significantly better dietary outcomes (Table 6). As shown in Table 6, this regular-use group exhibited improvements in moderation (*P*=.002), balance (*P*=.046), and overall nutrition score (*P*=.01). By contrast, those with irregular usage patterns (gaps >7 hours and variable timing) did not show improvements in these domains. These results indicate that consistent, frequent app engagement is associated with improved dietary behavior and cognitive outcomes. Notably, however, the irregular-use group reported a significant reduction in appetite loss symptoms (*P*=.04), while the regular-use group had minimal baseline appetite issues, leaving less room for improvement. No other QoL domains differed significantly by usage pattern.

**Table 6.** Comparison by app usage routine pattern.

Characteristics	Irregular usage pattern (>7hours, n=12)			Regular usage pattern (≤7 hours, n=12)		
	Before	After	*P *value	Before	After	*P* value
Nutrition						
Moderation	80.75 (8.86)^a^	80.00 (10.35)	.64	72.46 (19.42**)**	82.08 (15.07**)**	.002^b^
Balance	55.90 (19.24)	53.67 (13.16)	.69	62.60 (14.35**)**	66.92 (16.40**)**	.046^b^
Implementation	77.57 (13.49)	74.86 (11.68)	.70	79.43 (13.47)	77.12 (12.20)	.83
Nutritional score	72.02 (11.50)	70.04 (10.13)	.88	72.29 (10.19**)**	75.55 (11.36**)**	.01**^b^**
Quality of life						
Global health	53.48 (16.84)	51.39 (15.82)	.71	52.77 (11.43)	47.92 (14.27)	.76
Appetite loss	16.66 (22.47**)**	5.55 (12.97**)**	.04**^b^**	5.55 (12.96)	2.78 (9.62)	.33
Cognitive	74.98 (21.89)	76.39 (26.07)	.21	80.54 (13.90)	81.94 (18.06)	.12
Emotional	68.74 (23.87)	68.75 (22.51)	.36	80.55 (19.89)	74.31 (26.46)	.88
Social	84.72 (26.06)	70.83 (23.70)	.98	93.06 (16.60)	86.11 (8.58)	.97
Physical	84.45 (2.81)	83.89 (14.90)	.74	88.32 (9.90)	85.00 (14.25)	.73
Role	83.34 (25.61)	79.17 (20.26)	.85	87.50 (14.42)	87.50 (16.09)	.47
Nausea and vomiting	2.78 (6.50)	4.17 (10.36)	.50	4.17 (7.55)	1.39 (4.81)	.06
Constipation	24.98 (28.86)	25.00 (28.87)	.92	27.78 (34.33)	30.56 (36.12)	.60
Diarrhea	13.88 (17.15)	11.11 (16.41)	.76	8.32 (15.06)	5.55 (12.97)	.50
Pain	20.82 (17.58)	16.67 (15.89)	.289	13.90 (22.29)	13.89 (18.58)	.25
Dyspnea	19.43 (30.01)	19.44 (26.43)	.83	13.88 (22.28)	16.67 (22.47)	.92
Insomnia	33.32 (28.43)	36.11 (22.29)	.89	27.76 (19.24)	30.55 (22.29)	.90
Fatigue	40.73 (28.96)	39.81 (26.99)	.48	24.05 (14.84)	35.18 (21.63)	.98
Financial difficulties	13.88 (22.28)	22.22 (25.95)	.90	19.43 (30.01)	13.89 (17.16)	.83

a These values are expressed as mean (SD).

b Statistically significant results (*P*<.05).

### Exploratory Subgroup Analyses

Exploratory subgroup analyses revealed variations in intervention effects by age, sex, and cancer type (Tables S1-S3 in Multimedia Appendix 2). Among older participants (aged 60 years and older), significant improvements were observed in overall diet quality—reflected by higher Moderation subscores (*P*=.02)—and in cognitive functioning (*P*=.02), whereas younger participants showed no comparable changes. Female survivors also demonstrated a significant postintervention increase in Moderation (*P*=.04) that was not evident in males. In addition, survivors with non–breast cancers (eg, colorectal, gastric, or lung) exhibited a marked improvement in diet quality (*P*=.008) and greater reductions in appetite loss symptoms, while breast cancer survivors—who had higher baseline diet quality and minimal appetite complaints—showed little change. These subgroup findings suggest that age, sex, and cancer type may moderate the magnitude of benefit derived from the app-based dietary intervention.

## Discussion

### Principal Results

Although this study involved a short 4-week intervention, its primary purpose was to generate feasibility evidence and behavioral insights to inform the design of subsequent long-term trials, rather than to establish long-term efficacy. While some participants expressed an interest in weight control, the primary focus of this study was to improve dietary quality, appetite regulation, and overall well-being rather than to promote weight loss per se. In this 4-week pilot study, a mobile dietary self-management app for cancer survivors was associated with improvements in key nutrition-related behaviors and symptoms. Participants’ overall diet quality improved significantly, as shown by an increase in the Nutrition Quotient Moderation subscore (reflecting reduced consumption of unhealthy foods), and they reported a marked reduction in appetite loss symptoms. These positive changes are notable, given that many cancer survivors face challenges with inadequate dietary intake and appetite suppression during and after treatment.

The pronounced reduction in appetite loss may reflect not only improved nutritional intake but also partial alleviation of psychosocial factors (such as treatment-related stress or social isolation). The app provided personalized meal recommendations, diverse healthy recipes, and motivational messages, offering a multidimensional, individualized approach to stimulate appetite and encourage regular eating. This outcome aligns with survivorship nutrition guidelines, as the ACS encourages survivors to consume a variety of vegetables, fruits, and high-quality protein and to optimize the mealtime environment to help maintain or regain appetite—practices shown to support appetite recovery [24]. These findings illustrate that the guideline—implementation—effectiveness pathway proposed by the ACS can be operationalized through a digital platform, demonstrating that evidence-based nutrition guidance can translate into measurable behavioral and clinical improvements. By delivering tailored support consistent with these recommendations, the app may help survivors overcome appetite loss, a complex and common issue in cancer survivorship. Notably, maintaining a healthy weight is often recommended as part of recurrence prevention in breast, prostate, and endometrial cancer survivors. In this context, weight awareness or moderate control behaviors complement—rather than contradict—the goal of restoring appetite and balanced eating. A modest but significant improvement in pain symptoms was also noted among high-frequency app users, suggesting that consistent dietary self-monitoring may provide ancillary benefits for perceived physical well-being. Another key finding was a significant improvement in dietary moderation, indicating a shift away from unhealthy eating patterns (eg, frequent overeating, binge eating, or strong food aversions) toward more balanced dietary habits. This finding supports the emphasis of established dietary guidelines on moderation and limiting nutrient-poor, energy-dense foods. For example, ACS guidelines warn against Western-style diets high in fat and sugar and instead highlight the importance of high-fiber, low-fat diets with increased intake of plant-based foods while restricting processed, calorie-dense items [24]. Our results suggest that a digital intervention can facilitate these healthy dietary shifts in a real-world context, helping survivors adopt and sustain recommended eating behaviors. In addition, we observed an improvement in cognitive functioning scores among participants aged 60 years and older. Older cancer survivors are particularly susceptible to cancer-related cognitive impairment due to factors such as chemotherapy, chronic inflammation, sleep disturbances, and depression. The app included features that might support cognitive health, including suggestions for “brain-friendly” foods rich in omega-3 fatty acids and antioxidants, encouragement of regular meal routines, and tools for self-monitoring dietary intake. These components may have helped impose daily structure and improve mental clarity in older users. While this subgroup finding is exploratory, it raises the possibility that digital dietary interventions could confer ancillary cognitive benefits in older patients with cancer, warranting further investigation into the underlying mechanisms and durability of this effect. Importantly, our analysis found that greater engagement with the app was associated with larger improvements in outcomes. Participants who used the app more frequently, for longer total durations, and with more consistency (ie, fewer and shorter gaps between usage sessions) tended to achieve greater gains in healthy eating behaviors and QoL scores than those with lower usage. To our knowledge, this study is among the first in the cancer survivorship setting to capture such detailed app usage metrics (using time-stamped log data to define user sessions) and to link these engagement indicators to standardized nutrition and QoL outcomes. This analytic approach provides preliminary evidence that engagement may mediate the effectiveness of digital interventions. However, because this was an uncontrolled, short-term pilot, the observed relationships between engagement and outcomes should be interpreted as associative rather than causal, serving primarily as hypothesis-generating evidence for subsequent trials. This pilot was designed to assess short-term feasibility, adherence, and early symptom response rather than to verify long-term efficacy; the findings thus provide foundational data to inform the design and power estimation of future randomized controlled trials (RCTs).

Overall, these findings suggest that an app-based dietary program can serve as a comprehensive supportive tool that addresses not only survivors’ nutritional needs but also aspects of their psychological, cognitive, and behavioral health. The app’s capacity to deliver personalized nutrition guidance, promote beneficial behavior changes, and provide accessible support indicates that such digital interventions could be further refined and tailored to specific cancer types, age groups, and phases of survivorship for maximum benefit. Although the intervention period was limited to 4 weeks, this study was intentionally designed to align with the identified gap in the existing literature. Prior mHealth nutrition studies in cancer survivorship have been constrained not only by short follow-up durations but also by interventions narrowly focused on single components—such as calorie tracking or symptom logging—without examining how users engage with more comprehensive, behavior-oriented tools. In contrast, this study evaluated a multidimensional dietary intervention that integrates personalized nutrition guidance, lifestyle-related behavioral feedback, and symptom awareness, while explicitly quantifying real-world engagement patterns using objective log data. Within this framework, the 4-week time frame served as a necessary and methodologically appropriate first step to determine intervention feasibility, user adherence, and early behavioral signal detection—prerequisites for designing and justifying longer-term efficacy trials. Thus, rather than attempting to address long-term outcomes directly, this pilot study contributes to the literature by establishing whether the intervention demonstrates sufficient feasibility and engagement-driven responsiveness to warrant subsequent large-scale, long-term evaluation. This stepwise approach is consistent with recommended frameworks for the development and evaluation of complex digital health interventions.

### Comparison With Prior Work

Our findings are consistent with, and extend, prior research on mHealth nutrition interventions for cancer survivors. Previous pilot studies of app-based diet or weight management programs have reported improvements in dietary quality, weight maintenance, and patient-reported outcomes, and a recent systematic review of randomized trials concluded that mHealth interventions show promise for improving diet and physical activity in cancer survivor populations [12]. Similarly, the improvements observed in our study—such as enhanced dietary moderation and reduced symptom burden—mirror these earlier reports, reinforcing that app-based guidance can help meet survivors’ nutritional needs. Distinct from most earlier studies, this work quantitatively linked real-world app engagement metrics (frequency, duration, and continuity of use) to measurable changes in dietary and QoL outcomes. This analytic approach provides empirical groundwork for defining optimal adherence thresholds and intervention intensity in future trials. We add to the literature by demonstrating a direct link between participants’ engagement with the app and the magnitude of benefits achieved, a relationship that has seldom been examined in prior studies. Emerging evidence from other digital health studies also highlights the importance of sustained app engagement for successful behavior change and symptom management. For example, in a recent mobile weight loss program for breast cancer survivors, those who engaged more frequently with app features (such as logging meals or reading educational content) were significantly more likely to achieve clinically meaningful weight loss [25]. Likewise, Yunis et al [8] found that patients with cancer and caregivers had high adherence to a symptom-tracking app over 1 month, and their study demonstrated that detailed app use metadata (eg, time-stamped login information) can inform remote monitoring of patient engagement and well-being between clinic visits. Furthermore, adding interactive components to boost engagement can yield additional benefits: in an RCT, breast cancer survivors who participated in an app-based peer support community (vs using a tracking app alone) experienced significantly lower distress levels and increased physical activity [26].

Beyond these trials, recent systematic and observational evidence clarifies where this study advances the field (Table S1 in Multimedia Appendix 3). Shen et al [25] demonstrated that higher real-world engagement with a behavioral app predicted meaningful weight loss, supporting engagement as a key behavioral driver, although cognitive outcomes were not assessed. Wang et al [12] summarized 23 RCTs of mHealth diet and activity programs, confirming short-term benefits but highlighting limited mechanistic data and underrepresentation of older adults. Lu et al [27] found that nutritional interventions—including anti-inflammatory and fasting-mimicking diets and polyunsaturated fatty acids supplementation—can improve cognitive outcomes, yet these trials were investigator-driven rather than self-managed. Coro et al [28] qualitatively showed that survivors view diet as linked to mental clarity but struggle to maintain structured routines due to cognitive fog. Crowder et al [29] observed that higher monounsaturated fatty acids or saturated fatty acids ratios were associated with better short-term cognitive performance during chemotherapy, while McLeod et al [30] reported complex, context-dependent associations between Mediterranean diet adherence and cognitive outcomes in older adults. In contrast, this study uniquely integrates detailed engagement analytics with dietary, symptom, and QoL outcomes. By linking frequency, duration, and continuity of app use to improvements in diet quality and appetite loss, it provides concrete evidence supporting the engagement-outcome mechanism. Moreover, the inclusion of older survivors (aged 60 years and older) extends digital survivorship research into an age group seldom represented in prior studies and suggests potential cognitive and structural benefits associated with sustained app use. Taken together, these reports and our results underscore that maximizing patient engagement is vital for mHealth interventions to achieve their full potential in cancer care. Ensuring that survivors not only adopt an app but continue to use it actively and regularly appears to be a prerequisite for attaining meaningful improvements in health behaviors and QoL.

### Limitations

Several limitations of this pilot study must be acknowledged. First, this study had a modest sample size (n=24 completers), an uncontrolled single-arm design, and a short 4-week intervention, which limit generalizability and preclude causal inference. The short follow-up also meant that we could not determine the longevity of the observed behavior changes. Second, all outcomes were measured via self-reported questionnaires, introducing potential recall bias and social desirability bias. Third, participants received weekly check-in calls or text messages from study staff to encourage app use; this human support likely improved adherence and also confounds attribution of the outcomes to the app alone (ie, higher engagement may have been partly driven by these external prompts). Indeed, blended approaches that combine digital tools with human coaching are known to boost user engagement and can potentially amplify outcomes [31], so our study cannot disentangle the app’s independent effects from the influence of this added support. In addition, app-generated quantitative dietary intake data were not analyzed, as they were not prespecified efficacy end points and were variably incomplete due to early discontinuation of app use among noncompleters. Finally, while our sample included a range of ages and cancer types, the study was not powered to examine differences across subgroups; any exploratory observations of trends by age or diagnosis should therefore be interpreted with caution, as they were not the focus of this analysis. Another limitation is that detailed clinical and sociodemographic data—including employment status, comorbidities, and the exact interval since surgery—were not comprehensively collected. Although all participants were disease-free and within 5 years after curative surgery, the lack of these variables may limit contextual interpretation of outcomes. Despite these limitations, the completion rate (88.9%) and adherence rate (≥70%) exceeded predefined feasibility benchmarks, confirming the practicality and acceptability of the intervention. Notably, the absence of a control group and the short duration of follow-up mean that the durability of the behavior change is uncertain. This is a common issue in early mHealth studies, and a recent review highlighted that evidence for the long-term efficacy of nutrition apps in cancer care remains inconclusive [32]. Accordingly, our positive findings should be viewed as preliminary and hypothesis-generating. Although quantitative food record data were partially collected, response completeness was insufficient for reliable analysis. Therefore, this pilot prioritized a qualitative dietary assessment tool that captures overall meal quality and behavioral changes, which better reflect the app’s behavioral focus and feasibility objectives. Future large-scale studies will integrate quantitative nutrient analyses for comprehensive validation.

### Future Directions

Building on this pilot, future research should validate and expand these findings using more robust study designs. In particular, larger RCTs are needed to confirm the relationships observed between app engagement and outcomes and to determine whether actively boosting user engagement can causally improve nutritional status or clinical results. Such trials should also include longer follow-up periods to assess whether improvements in diet and QoL are sustained over time, and they should incorporate objective outcome measures (eg, weight change or nutritional biomarkers) alongside self-reports for a more comprehensive evaluation of the intervention’s impact. Another priority for future interventions is to leverage engagement-tailored personalization. Our data suggest that users vary in how actively and consistently they use the app, and these usage patterns likely influence the benefits gained. Tailoring the program to an individual’s engagement profile could amplify its effectiveness. For example, the app could use just-in-time adaptive features such as real-time alerts or motivational feedback when a lapse in use is detected, or dynamically adjust the frequency of prompts and content based on each user’s behavior. By using analytics to identify when a participant is at risk of disengaging, the intervention could deliver timely, personalized support (such as encouragement from a coach, simplified tasks, or new content to recapture interest). Such adaptive personalization may help prevent drop-off and maximize the “dose” of the intervention each participant receives, ultimately leading to better outcomes. Additionally, future efforts should integrate mHealth dietary apps into standard survivorship care. Rather than functioning in isolation, apps like ours can be embedded within oncology nutrition services and survivorship care plans to extend support beyond the hospital setting. For instance, integrating the app with routine follow-up visits or with electronic health records could enable nutritionists and clinicians to remotely monitor patients’ dietary logs and symptoms and intervene earlier when issues arise [8]. Care teams might receive automated summaries of a survivor’s app activity or alerts if a patient’s engagement or nutritional status falls below a certain threshold. This type of health system integration would facilitate a more continuous model of care. By providing an accessible, scalable platform for dietary self-management that remains connected to professional support, a validated nutrition app could reach survivors who otherwise might not receive ongoing nutritional guidance.

### Conclusions

This pilot study demonstrates the feasibility of a mobile app–based dietary intervention for cancer survivors and provides preliminary evidence of its benefits. Participants achieved improvements in diet-related measures and QoL, particularly in those domains (such as healthy eating habits and symptom relief) where the app’s guidance was most directly applied. Notably, greater user engagement was associated with greater improvements, highlighting the critical role of sustained engagement in the success of digital health interventions. While these results require confirmation in controlled trials, they underscore the potential of personalized, engagement-driven mHealth tools to support the long-term health and well-being of cancer survivors.

## Supplementary material

10.2196/79215 Multimedia Appendix 1 Comparison of nutrition and quality of life before and after app use by sex.

10.2196/79215 Multimedia Appendix 2 Comparison of nutrition and quality of life before and after app use by age group.

10.2196/79215 Multimedia Appendix 3 Systematic review.

10.2196/79215 Checklist 1 CONSORT (Consolidated Standards of Reporting Trials) checklist.

## Abbreviations

ACS: American Cancer Society
CONSORT: Consolidated Standards of Reporting Trials
QLQ-C30: European Organization for Research and Treatment of Cancer Quality of Life Questionnaire Core 30
mHealth: mobile health
NQ-2021: Nutrition Quotient for Adults
QoL: quality of life
RCT: randomized controlled trial
STROBE: Strengthening the Reporting of Observational Studies in Epidemiology
