# Supplementary material for: Impact of a Mobile Nutrition App on Dietary Outcomes in Cancer Survivors: Pilot Feasibility Study
Source: JMIR Cancer. 2026 Mar 31;12:e79215. doi: 10.2196/79215 (PMC13038178; doi:10.2196/79215)
Supplement: Multimedia Appendix 1 [file cancer-v12-e79215-s001.docx]

Table S1. Comparison of nutrition and quality of life before and after app use by sex.

|  | | **14 days** | | | **21 days** | | | **28 days** | | |
| --- | --- | --- | --- | --- | --- | --- | --- | --- | --- | --- |
|  |  | **Before** | **After** | ***P*** | **Before** | **After** | ***P*** | **Before** | **After** | ***P*** |
| **Nutrition** | Moderation | 75.11 ± 17.51 | 80.58 ± 13.74 | .018 | 72.71 ± 20.14 | 77.99 ± 14.28 | .042 | 73.36 ± 18.34 | 79.62 ± 7.77 | .148 |
|  | Balance | 63.72 ± 16.06 | 65.46 ± 13.26 | .137 | 62.47 ± 15.32 | 65.71 ± 14.25 | .160 | 55.8 ± 15.07 | 65.29 ± 15.75 | .023 |
|  | Implementation | 80.55 ± 13.44 | 76.78 ± 11.23 | .961 | 84.29 ± 13.77 | 77.5 ± 12.8 | .984 | 83.83 ± 16.09 | 75.95 ± 12.73 | .977 |
|  | Nutritional score | 73.87 ± 10.05 | 74.53 ± 9.91 | .264 | 74.27 ± 10.55 | 74.11 ± 11.34 | .483 | 72.28 ± 10.61 | 73.85 ± 10.36 | .234 |
| **Life quality** | Global health | 55.72 ± 9.49 | 50.0 ± 14.59 | .878 | 56.05 ± 10.61 | 53.03 ± 15.93 | .541 | 52.36 ± 9.27 | 48.81 ± 15.54 | .542 |
|  | Appetite loss | 8.32 ± 14.89 | 2.08 ± 8.33 | .065 | 6.05 ± 13.47 | - | .079 | 9.51 ± 16.25 | - | .079 |
|  | Cognitive | 83.32 ± 10.53 | 84.37 ± 11.33 | .132 | 81.81 ± 11.66 | 83.33 ± 10.54 | .189 | 80.94 ± 11.48 | 80.95 ± 11.5 | .294 |
|  | Emotional | 80.73 ± 14.81 | 76.04 ± 13.56 | .915 | 80.3 ± 16.35 | 74.24 ± 15.12 | .917 | 73.81 ± 16.25 | 70.24 ± 15.1 | .656 |
|  | Social | 92.71 ± 14.87 | 79.17 ± 18.76 | .996 | 90.91 ± 17.26 | 78.79 ± 19.85 | .986 | 85.71 ± 20.25 | 78.57 ± 20.89 | .946 |
|  | Physical | 87.49 ± 11.64 | 85.0 ± 14.3 | .748 | 87.86 ± 11.87 | 86.06 ± 15.04 | .472 | 84.74 ± 10.69 | 87.62 ± 7.13 | .047 |
|  | Role | 88.54 ± 14.54 | 86.46 ± 15.18 | .745 | 89.4 ± 15.39 | 89.39 ± 13.48 | .660 | 85.73 ± 17.8 | 90.48 ± 13.11 | .138 |
|  | Nausea, vomiting | 2.09 ± 5.7 | 1.04 ± 4.17 | .138 | 3.04 ± 6.76 | 1.52 ± 5.03 | .138 | 4.77 ± 8.15 | 0.0 ± 0.0 | .079 |
|  | Constipation | 22.91 ± 29.11 | 22.92 ± 26.44 | .622 | 24.24 ± 33.64 | 30.3 ± 27.71 | .850 | 38.09 ± 35.64 | 38.09 ± 23.0 | .706 |
|  | Diarrhea | 12.49 ± 16.65 | 10.42 ± 15.96 | .841 | 6.05 ± 13.47 | 6.06 ± 13.48 | .921 | 4.76 ± 12.59 | 4.76 ± 12.6 | .841 |
|  | Pain | 17.71 ± 22.34 | 15.63 ± 18.73 | .193 | 18.19 ± 25.23 | 19.7 ± 20.84 | .376 | 26.2 ± 28.64 | 21.43 ± 20.89 | .137 |
|  | Dyspnea | 12.49 ± 20.63 | 16.67 ± 24.34 | .958 | 15.15 ± 22.92 | 18.18 ± 22.92 | .921 | 14.27 ± 17.8 | 19.05 ± 17.82 | .971 |
|  | Insomnia | 29.14 ± 16.67 | 31.25 ± 22.67 | .930 | 27.25 ± 20.1 | 27.27 ± 25.03 | .764 | 28.56 ± 23.01 | 33.33 ± 27.22 | .830 |
|  | Fatigue | 22.89 ± 16.45 | 31.25 ± 18.69 | .992 | 19.17 ± 16.53 | 27.27 ± 16.75 | .982 | 26.96 ± 14.12 | 33.33 ± 12.83 | .891 |
|  | Financial difficulties | 10.41 ± 15.94 | 18.75 ± 20.97 | .995 | 9.08 ± 15.55 | 18.18 ± 22.92 | .980 | 14.27 ± 17.8 | 19.05 ± 17.82 | .971 |
