# Supplementary material for: Impact of a Mobile Nutrition App on Dietary Outcomes in Cancer Survivors: Pilot Feasibility Study
Source: JMIR Cancer. 2026 Mar 31;12:e79215. doi: 10.2196/79215 (PMC13038178; doi:10.2196/79215)
Supplement: Multimedia Appendix 2 [file cancer-v12-e79215-s002.docx]

Table S2. Comparison of nutrition and quality of life before and after app use by age group.

|  |  | **Under 60 years (N=13)** | | | **Over 60 years (N=11)** | | |
| --- | --- | --- | --- | --- | --- | --- | --- |
|  |  | **Before** | **After** | ***P*** | **Before** | **After** | ***P*** |
| **Nutrition** | Moderation | 77.05 ± 7.14 | 80.45 ± 10.11 | .188 | 76.08 ± 21.92 | 81.74 ± 15.7 | .019 |
|  | Balance | 52.5 ± 17.35 | 55.72 ± 16.46 | .122 | 67.22 ± 13.06 | 65.7 ± 14.4 | .382 |
|  | Implementation | 77.29 ± 15.46 | 74.31 ± 13.9 | .801 | 79.93 ± 10.54 | 77.97 ± 8.78 | .854 |
|  | Nutritional score | 69.78 ± 11.69 | 70.58 ± 11.63 | .393 | 74.96 ± 8.94 | 75.42 ± 9.85 | .289 |
| **Life quality** | Global health | 53.21 ± 17.53 | 54.49 ± 15.07 | .297 | 53.02 ± 9.33 | 43.94 ± 12.96 | .949 |
|  | Appetite loss | 12.82 ± 21.68 | 5.13 ± 12.52 | .070 | 9.08 ± 15.55 | 3.03 ± 10.05 | .138 |
|  | Cognitive | 74.35 ± 23.17 | 74.36 ± 28.56 | .318 | 81.8 ± 8.97 | 84.85 ± 8.99 | .021 |
|  | Emotional | 64.73 ± 23.61 | 64.1 ± 29.34 | .342 | 86.36 ± 14.07 | 80.3 ± 12.51 | .946 |
|  | Social | 87.18 ± 25.59 | 78.21 ± 25.8 | .947 | 90.91 ± 17.26 | 78.79 ± 18.39 | .987 |
|  | Physical | 87.69 ± 9.76 | 87.69 ± 8.09 | .541 | 84.85 ± 13.36 | 80.61 ± 18.96 | .885 |
|  | Role | 88.47 ± 15.77 | 83.33 ± 20.41 | .897 | 81.82 ± 25.21 | 83.33 ± 16.67 | .432 |
|  | Nausea, vomiting | 5.14 ± 8.02 | 3.85 ± 9.99 | .246 | 1.52 ± 5.04 | 1.52 ± 5.03 | .327 |
|  | Constipation | 28.19 ± 29.96 | 30.77 ± 34.59 | .951 | 24.24 ± 33.64 | 24.24 ± 30.15 | .458 |
|  | Diarrhea | 12.81 ± 16.86 | 7.69 ± 14.62 | .339 | 9.08 ± 15.55 | 9.09 ± 15.57 | .958 |
|  | Pain | 16.67 ± 16.66 | 14.1 ± 14.98 | .162 | 18.18 ± 24.1 | 16.67 ± 19.72 | .368 |
|  | Dyspnea | 10.25 ± 16.0 | 10.26 ± 16.01 | .977 | 24.24 ± 33.64 | 27.27 ± 29.13 | .802 |
|  | Insomnia | 35.88 ± 28.75 | 38.46 ± 22.96 | .852 | 24.22 ± 15.55 | 27.27 ± 20.1 | .933 |
|  | Fatigue | 36.73 ± 23.74 | 37.61 ± 26.27 | .580 | 27.25 ± 24.53 | 37.37 ± 22.37 | .970 |
|  | Financial difficulties | 20.51 ± 32.03 | 17.95 ± 25.88 | .584 | 12.11 ± 16.8 | 18.18 ± 17.41 | .988 |

Table S2. Comparison of nutrition and quality of life before and after app use by cancer type.

|  | | **Breast cancer (N=11)** | | | **Other cancer (N=13)** | | |
| --- | --- | --- | --- | --- | --- | --- | --- |
|  |  | **Before** | **After** | ***P*** | **Before** | After | ***P*** |
| **Nutrition** | Moderation | 81.37 ± 5.05 | 81.7 ± 9.21 | .382 | 72.57 ± 19.81 | 80.49 ± 15.4 | .008 |
|  | Balance | 57.46 ± 15.44 | 62.14 ± 14.8 | .103 | 60.77 ± 18.62 | 58.73 ± 17.46 | .554 |
|  | Implementation | 81.49 ± 11.66 | 76.27 ± 11.86 | .908 | 75.97 ± 14.37 | 75.75 ± 12.1 | .594 |
|  | Nutritional score | 74.24 ± 7.3 | 73.66 ± 10.83 | .585 | 70.39 ± 12.83 | 72.06 ± 11.34 | .188 |
| **Life quality** | Global health | 53.03 ± 16.36 | 52.27 ± 13.49 | .476 | 53.2 ± 12.53 | 47.43 ± 16.1 | .886 |
|  | Appetite loss | 3.03 ± 10.04 | 0.0 ± 0.0 | .159 | 17.94 ± 22.0 | 7.69 ± 14.62 | .055 |
|  | Cognitive | 72.72 ± 22.68 | 75.76 ± 27.25 | .207 | 82.03 ± 12.65 | 82.05 ± 17.3 | .092 |
|  | Emotional | 70.45 ± 26.71 | 73.49 ± 26.3 | .076 | 78.2 ± 18.17 | 69.87 ± 23.21 | .973 |
|  | Social | 81.82 ± 29.29 | 80.3 ± 27.71 | .710 | 94.87 ± 10.51 | 76.92 ± 17.4 | .996 |
|  | Physical | 86.65 ± 10.75 | 89.09 ± 7.47 | .070 | 86.16 ± 12.3 | 80.51 ± 17.52 | .988 |
|  | Role | 87.88 ± 15.06 | 87.88 ± 21.2 | .458 | 83.34 ± 24.52 | 79.49 ± 15.45 | .871 |
|  | Nausea, vomiting | 4.55 ± 7.80 | 1.52 ± 5.03 | .051 | 2.57 ± 6.27 | 3.85 ± 9.99 | .500 |
|  | Constipation | 33.32 ± 36.51 | 30.3 ± 31.46 | .667 | 20.51 ± 25.6 | 25.64 ± 33.76 | .850 |
|  | Diarrhea | 3.03 ± 10.04 | 3.03 ± 10.05 | .841 | 17.93 ± 17.28 | 12.82 ± 16.88 | .570 |
|  | Pain | 19.7 ± 23.36 | 18.18 ± 18.94 | .300 | 15.38 ± 17.29 | 12.82 ± 15.45 | .257 |
|  | Dyspnea | 9.08 ± 15.55 | 9.09 ± 15.57 | .958 | 23.07 ± 31.58 | 25.64 ± 27.74 | .850 |
|  | Insomnia | 33.32 ± 29.82 | 36.36 ± 23.36 | .862 | 28.18 ± 18.49 | 30.77 ± 21.35 | .926 |
|  | Fatigue | 32.31 ± 26.05 | 31.31 ± 24.76 | .480 | 32.45 ± 23.33 | 42.73 ± 23.06 | .976 |
|  | Financial difficulties | 24.24 ± 33.64 | 15.15 ± 22.92 | .393 | 10.25 ± 16.0 | 20.51 ± 21.68 | .992 |

Table S3. Comparison of nutrition and quality of life before and after app use by sex.

|  | | **Male (N=7)** | | | **Female (N=17)** | | |
| --- | --- | --- | --- | --- | --- | --- | --- |
|  |  | **Before** | **After** | ***P*** | **Before** | **After** | ***P*** |
| **Nutrition** | Moderation | 76.68 ± 21.0 | 84.59 ± 13.16 | .113 | 76.57 ± 13.17 | 79.58 ± 12.6 | .044 |
|  | Balance | 62.6 ± 15.37 | 63.2 ± 8.33 | .148 | 57.87 ± 17.82 | 59.1 ± 18.4 | .322 |
|  | Implementation | 72.48 ± 10.31 | 74.39 ± 10.41 | .358 | 80.98 ± 13.74 | 76.65 ± 12.48 | .941 |
|  | Nutritional score | 70.78 ± 10.96 | 74.09 ± 7.88 | .055 | 72.73 ± 10.78 | 72.26 ± 12.1 | .609 |
| **Life quality** | Global health | 52.37 ± 10.46 | 48.81 ± 12.2 | .663 | 53.43 ± 15.61 | 50.0 ± 16.14 | .775 |
|  | Appetite loss | 14.27 ± 17.8 | 4.76 ± 12.6 | .138 | 9.8 ± 19.59 | 3.92 ± 11.07 | .070 |
|  | Cognitive | 88.07 ± 8.15 | 88.09 ± 12.6 | .168 | 73.52 ± 19.58 | 75.49 ± 24.38 | .153 |
|  | Emotional | 79.76 ± 11.63 | 79.76 ± 10.6 | .416 | 72.54 ± 25.48 | 68.14 ± 27.52 | .766 |
|  | Social | 92.86 ± 13.11 | 80.95 ± 17.82 | .967 | 87.25 ± 24.67 | 77.45 ± 24.25 | .981 |
|  | Physical | 88.59 ± 12.59 | 84.76 ± 13.17 | .979 | 85.48 ± 11.11 | 84.31 ± 15.08 | .500 |
|  | Role | 88.1 ± 15.84 | 83.33 ± 16.67 | .821 | 84.32 ± 22.4 | 83.33 ± 19.54 | .622 |
|  | Nausea, vomiting | 2.39 ± 6.31 | 0.0 ± 0.0 | .159 | 3.93 ± 7.3 | 3.92 ± 9.37 | .298 |
|  | Constipation | 19.04 ± 26.23 | 14.29 ± 26.23 | .207 | 29.4 ± 33.09 | 33.33 ± 33.33 | .926 |
|  | Diarrhea | 23.79 ± 16.25 | 19.05 ± 17.82 | .760 | 5.88 ± 13.09 | 3.92 ± 11.07 | .500 |
|  | Pain | 14.29 ± 14.98 | 7.14 ± 8.91 | .113 | 18.63 ± 21.95 | 18.63 ± 18.52 | .439 |
|  | Dyspnea | 9.51 ± 16.25 | 14.29 ± 26.23 | .910 | 19.6 ± 29.01 | 19.61 ± 23.74 | .848 |
|  | Insomnia | 28.54 ± 12.59 | 28.57 ± 23.0 | .882 | 31.36 ± 27.57 | 35.29 ± 21.96 | .922 |
|  | Fatigue | 31.71 ± 11.87 | 38.09 ± 20.14 | .891 | 32.66 ± 27.91 | 37.25 ± 26.04 | .883 |
|  | Financial difficulties | 14.27 ± 17.8 | 19.05 ± 17.82 | .971 | 17.64 ± 29.15 | 17.65 ± 23.91 | .737 |
