# Supplementary material for: Impact of a Mobile Nutrition App on Dietary Outcomes in Cancer Survivors: Pilot Feasibility Study
Source: JMIR Cancer. 2026 Mar 31;12:e79215. doi: 10.2196/79215 (PMC13038178; doi:10.2196/79215)
Supplement: Multimedia Appendix 3 [file cancer-v12-e79215-s003.docx]

Table S3. Systematic review.

| **Study (Year, Journal)** | **Design & Population** | **Intervention / Exposure** | **Key Findings** |
| --- | --- | --- | --- |
| Wang et al.[12] | Systematic review; 23 RCTs in cancer survivors | Digital diet / activity interventions | Diet & PA improved; QoL partly better |
| Shen et al.[25] | Single-arm, 26 wk; 31 breast cancer survivors | Mobile weight-management app (Noom®) | −4.8 kg (−5.6%); 35.5% ≥5% loss; higher app engagement ↔ weight loss |
| Lu et al.[27] | Systematic review; 59 RCTs in adults with cancer | Nutritional support; anti-inflammatory / FMD diets; PUFA supplements | Several interventions improved cognition |
| Coro et al.[28] | Qualitative; cancer survivors with CRCI | Diet–cognition perceptions | Diet linked to mental clarity; CRCI hinders planning & eating |
| Crowder et al.[29] | Prospective cohort; 96 patients during chemotherapy | Diet indices (MUFA/SFA ratio) | Higher MUFA/SFA ↔ better short-term cognition |
| McLeod et al.[30] | Cross-sectional; 2,450 adults (385 survivors) | Mediterranean diet adherence (aMed) | No overall cancer–cognition association; interaction with aMed |
| Our study | Single-arm pilot; 4 wk; 24 cancer survivors (≥60 included) | Mobile nutrition app with diet logging & analytics | ↑Diet moderation; Appetite loss; QoL; engagement ↔ improvement |
